# A concept study on non-targeted screening for chemical contaminants in food using liquid chromatography–mass spectrometry in combination with a metabolomics approach

**DOI:** 10.1007/s00216-012-6506-5

**Published:** 2012-11-01

**Authors:** Erik Tengstrand, Johan Rosén, Karl-Erik Hellenäs, K. Magnus Åberg

**Affiliations:** 1 Department of Analytical Chemistry, Stockholm University, 10691 Stockholm, Sweden; 2 National Food Administration, Box 622, 75126 Uppsala, Sweden

**Keywords:** Chemometrics/statistics, Foods/beverages, HPLC, Mass spectrometry/ICP-MS

## Abstract

A generic method to screen for new or unexpected contaminants at ppm levels in food has been developed. The method comprises an acidic acetonitrile extraction, detection with ultra-high-pressure liquid chromatography coupled to electrospray ionisation time-of-flight mass spectrometry and statistical evaluation using a metabolomics approach comparing suspected contaminated food with uncontaminated foods. The method was tested for 26 model contaminants from 100 μg/g down to 0.4 μg/g in three brands of fresh orange juice. Blinded statistical evaluation revealed signals from all added contaminants detectable by liquid chromatography–electrospray ionisation using positive ionisation mode, while only two false-positive signals were reported. The method is primarily intended to be used for investigation of food samples suspected to be contaminated with unknown substances. Additionally it could be used to continuously monitor for appearance of new food contaminants as a complement to the specific targeted analysis that is today’s foundation of food safety analysis.

## Introduction

In regular food analysis, a targeted approach is generally used, i.e. the analytes of interest are selected before making measurements. Commonly the methods aim to monitor ppb levels of the chosen compounds. The reason for monitoring very low levels are today’s food policies aiming to guarantee minimal risks for any toxic effect after life-long exposure, using safety margins of orders of magnitude [1]. The problem with targeted methods is that chemicals which are not initially anticipated are not detected regardless of how high their concentration might be.

Using modern techniques, such as time-of-flight mass spectrometry (TOF-MS) combined with ultra-high-pressure liquid chromatography (UHPLC), data on thousands of analytes not selected beforehand can be generated. This instrumentation is often used in metabolomics to characterise highly complex samples. The biomarker approach of metabolomics involves statistically analysing the data to single out compounds that increase or decrease in disease or treatments. The approach might also be used in food analysis for comparisons between different samples of a specific food type, e.g. in order to detect new or unexpected food contaminants, or reveal time trends. However, to date, there have only been a few reports of using a metabolomics approach in food safety [2–4]. Foodomics has been defined as the use of ‘omics’ techniques applied within the domains of food and nutrition. The use of mass spectrometry within foodomics has recently been reviewed, including applications such as safety evaluation of GM foods, traceability and origin of foods [5].

One example of an incident involving an unexpected contaminant was when melamine was used as an adulterant in milk in China. Because melamine was not expected in milk, it was not a regulated compound and hence not tested for in food safety analyses. Melamine is not particularly toxic [6]. However, since such high concentrations were used (as high as 0.47 %) [7], its use caused toxic effects and even fatal outcomes. This incident suggests that a non-targeted approach to food control would be useful as a complement to the regular targeted detection methods. While non-targeted analysis may be less sensitive, its broader scope of analytes provides the possibility to pick up unexpected contaminants.

In this paper, we present a method that uses UHPLC–TOF-MS and a metabolomics approach to screen for deviant chemical compounds in food samples, and provide compound specific signals that can be used for further investigations of the chemical identity of the possible contaminants detected. The approach is based on the idea that only a limited number of compounds are analysed in regular food control, while a vast number of contaminants are possible. The methodology is primarily suggested to be applicable for suspected food samples, but the scope might also be widened to regular food testing. The new method was tested in a blinded study where different brands of orange juice have been spiked with model contaminants at levels well below acute toxicity.

## Experimental

Sampling, generation of spiked samples, extraction and UHPLC–TOF-MS analysis were performed at the National Food Administration, Sweden. Cartons of fresh orange juice of three brands, two packages of each, were purchased from a local food market. One sample per brand was spiked with model contaminants (either 11 mycotoxins, 18 pesticides or one pharmaceutical chemical) as shown in Table 1. The spiked samples were obtained by adding standard solutions (100 μL) to 1 mL of sample. These standard solutions were all methanol based and contained either seven mycotoxins at 4 μg/mL, or 18 pesticides at 25 μg/mL, or one pharmaceutical (sulfadoxin) at 1,000 μg/mL. Details of the added chemicals are found in Table 2. The spiked samples were mixed for 15 s by means of a standard vortex device and left in room temperature for 30 min to allow for equilibration prior to extraction. Spiked and blank samples (1 mL) were mixed with water (1 mL). Acetonitrile (6 mL) was added and the samples were vigorously agitated (1 min). The extracts were subjected to centrifugation (Heraeus Multifuge 3; 3,000×*g*, 10 °C, 10 min) and the supernatant was extracted and stored at 5 °C until analysis by UHPLC–TOF. The chromatographic system was based on a method developed by Owens et al. [8], which has been shown to be an efficient approach for multi-residue pesticide screening. The UHPLC column was an Acclaim RSLC 120 (C18, 2 μm, 2.1 × 100 mm from Dionex) maintained at 30 °C. A mobile phase gradient from 11 % to 100 % methanol in water, with 5 mM ammonium formate and 0.02 % formic acid, was used as well as a flow gradient from 200 to 450 μL/min (see Table 3 for details). Bottle A contained 0.315 g ammonium formate (>99 %, Fluka), 900 mL water from a Millipore purification system (MilliQ-Integral), 100 mL methanol (LiChrosolve, Merck) and 200 μL formic acid (pa, Merck). Bottle B contained 0.315 g ammonium formate, 1,000 mL methanol and 200 μL formic acid. The UHPLC instrument was a Dionex UltiMate 3000RS, and the injected sample volume was 2 μL. TOF-MS measurements were made using a Bruker QTOF ‘maXis’ set at a resolution of 40,000 (full width at half maximum at *m*/*z* = 922, called focus mode) or 20,000 (non-focus mode), and data were collected from *m*/*z* = 50 to 800 in positive mode at 2 Hz. The *m*/*z* calibration of the instrument was performed in three steps according to the manufacturer’s instructions: an initial rough calibration using Na(NaCOOH)_n_ clusters before any spectra were recorded, a fine calibration using the same calibrant before each analysis and finally a lock-mass calibration for every scan using methyl stearate at *m*/*z* = 299.2945. The TOF-MS settings were as follows: end plate offset −500 V, capillary −4,500 V, nebuliser 2.4 bar, dry gas 8.0 L/min, dry temperature 190 °C, funnel radiofrequency (RF) 400 V peak-to-peak (Vpp), multipole RF 200 Vpp, ion cooler RF 35 Vpp, transfer time 37 μs and pre-pulse storage time 5.0 μs.

**Table 1:** The experimental design

Brand	Package no.	Sample	Addition	Run order
Brämhult^a^	1	1		2, 17
Brämhult	1	2		8
Brämhult	1	3	Mycotoxins (7 at 0.4 μg mL^−1^)	14
Brämhult	2	1		5
Brämhult	2	2		11
Tropicana	1	1		3
Tropicana	1	2		9
Tropicana	1	3	Pesticides (18 at 2.5 μg mL^−1^)	15
Tropicana	2	1		6
Tropicana	2	2		12
Willys	1	1		4
Willys	1	2		10
Willys	1	3	Pharmaceutical (one at 100 μg mL^−1^)	16
Willys	2	1		7
Willys	2	2		13
Water		1	Blank	1
Water		2	Blank	18
Water		3	Blank	19

Replicates were made for the entire workup. Repeat injections of the prepared samples were performed on a different day with an identical run order

^a^The sample was injected twice as an instrument replicate

**Table 2:** Elution times and mass-to-charge ratios of peaks identified by the statistical evaluation as positive findings, and their attribution to spiked compounds or unknown (false positive)

Compound	Elution time	*m*/*z* assigned		
		[M+H]^+^	No. of additional isotopes and fragments	Adducts
Mycotoxins (0.4 μg mL^−1^)				
Aflatoxin G2	5.76	331.0815	1	[M+Na]^+^
Aflatoxin G1	6.09	329.0646	1	
Aflatoxin B2	6.44	315.0861	1	[M+Na]^+^
Aflatoxin B1	6.78	313.0708	1	
Diacetoxyscirpenol	6.86		[M+Na]^+^ at 389.1561, [M+K]^+^ [M+NH_4_]^+^	
T2 toxin	9.00	489.2099	0	
Sterigmatocystin	10.27	325.0709	1	[M+Na]^+^
False-positive peak	14.71	315.2301	0	
Pharmaceutical (100 μg mL^−1^)				
Column bleed artefact	4.72	226.9516	0	
Sulfadoxin	4.78	311.0844	10	[M+Na]^+^ [M+K]^+^ [M+2H]^2+^
Pesticides (2.5 μg mL^−1^)				
Acephate	3.12	184.0190	3	[M+Na]^+^
Omethoate	3.35	214.0303	4	[M+Na–H_2_O]^+^
Impurity	4.40	272.0716	1	
Dimethoate	5.28	230.0069	8	
Paraoxonmethyl	6.39	248.0323	2	[M+Na]^+^
Dichlorvos	7.02	220.9536	3	
Fenthion-sulfone	7.59	311.0170	4	[M+NH_4_]^+^ [M+Na]^+^
Atrazine	8.19	216.1015	4	
Metalaxyl	8.23	280.1586	6	[M+Na]^+^
Methidathion	8.74	302.9687	3	
Impurity	9.46	279.0270	0	
Triadimefon	9.71	294.1004	1	[M+Na]^+^
Fenarimol	10.16		Isotopes at 333.0366 and 335.035 were detected	
Tebuconazole	10.89		[M+Na]^+^ were detected at 330.1344 and 331.1370	
Chlorfenvinfos	10.97	358.9770	12	[M+Na]^+^
Diazinon	11.02	305.1092	2	
Fenthion	11.03	279.0282	2	[M+Na]^+^ [M+K]^+^
Propiconazol	11.07	342.0771	3	[M+Na]^+^
Prochloraz	11.23	376.0379	9	[M+Na]^+^
Impurity	11.28	362.9714	0	
Ethion	12.44	384.9952	10	[M+Na]^+^ [M+K]^+^

*m*/*z* ratios are given for the main ion of each peak; other isotopes and fragments are only reported in number. Inspection of the raw data revealed additional peaks in some cases, but these are not included in the table

**Table 3:** The gradients used in the chromatographic system

Time (min)	Flow (mL/min)	%B
0.0	0.20	1.0
0.1		1.0
1.0	0.20	
3.0		39.0
14.0	0.40	99.9
16.0	0.48	99.9
16.1		1.0
19.0	0.48	
19.1	0.20	

Linear gradients were used between set points

### Data processing

Line spectra were exported as files in netCDF format and examined using TracMass [9] at Stockholm University. The data analysis was performed blind, i.e. the people who performed the data analysis were not aware of which compounds had been added to the samples, nor how many.

### Peak detection

Peak detection was performed using a zero-area filter [10]. In brief, a chromatogram was convolved with the second derivative of a Gaussian model peak. Maxima in the filtered chromatogram were identified as potential peaks and compared to the noise level, which was estimated locally for each data point. The noise was extracted by smoothing the chromatogram with a Gaussian function and taking the difference between the original and smoothed chromatogram. The noise level was then estimated by calculating a locally weighted standard deviation for each data point. Maxima with signal-to-noise ratios greater than 30 were accepted as peaks.

The details of the peak detection were the following: the peak width at half maximum was approximately 4 s, *σ* for the zero-area filter was 1.5 s and *σ* for the smoothing Gaussian was 0.38 s.

### Peak alignment

Peak alignment is used to assign corresponding peaks to the same analyte in different samples. The observed retention times and mass-to-charge ratios of a particular analyte varied between samples. For screening, this creates a non-trivial alignment problem [11, 12].

To align the peaks, we used clustering followed by the generalised fuzzy Hough transform (GFHT) to resolve ambiguities. Clustering was performed by Delaunay triangulation after rescaling the retention time and mass by their approximate uncertainties. Delaunay triangulation creates a network of connections. Connections longer than one in the scaled mass–time space were deleted to yield a cluster structure. The clusters could contain more than one peak from one or more samples resulting in an ambiguity, e.g. this can occur for closely eluting peaks with the same *m*/*z* ratio. Ambiguous clusters were resolved into two or more clusters using GFHT in an analogous manner to the method described previously for NMR [13]. A correctly aligned table of peak intensities makes the subsequent data analysis more powerful.

### Identifying contaminants

Peaks that were only evident in spectra of the spiked samples were attributed to the contaminants. Of these, only peaks that were found in both replicates were accepted. These criteria were used when comparing the spiked sample with samples from the other brands. The motivation of these criteria is that in an investigation of an incident, mainly compounds that are not naturally occurring in foodstuffs should be investigated.

## Results and discussion

All added model compounds were detected. Potential contaminants that are incompatible with the analysis method will not be detected. This problem can be solved by performing complementary analyses using different methods. We used reverse phase liquid chromatography coupled to time-of-flight mass spectrometry, but any chromatographic method coupled to mass spectrometry could be used with the proposed approach. No clean-up step was used to avoid discriminating against some substances. We believe this to be important when looking for unknown compounds, even though the lack of clean-up may lower the robustness of the analytical method.

**Fig. 1:**
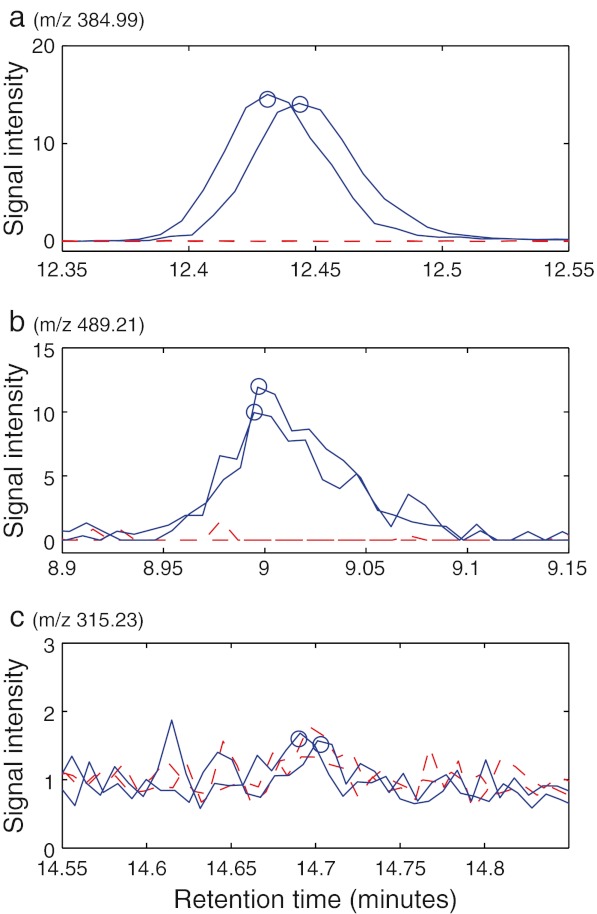
Chromatograms for three peaks attributed to ethion (**a**, moderate intensity); T2 toxin, which had the weakest intensity of all the model compounds (**b**); and a false positive (**c**). The *blue lines* indicate chromatograms where the peaks were detected, whereas the *red dashed lines* show two chromatograms where the peaks were not found

When comparing the spiked samples to samples from the other brands, the method falsely reported peaks from potential contaminants at a total of seven retention times. Of the seven positive results, two were in the dead time region and were not investigated further because of the high chemical noise, one was an artefact from column bleed that was detected as a peak, three were impurities in the standard solutions of the model compounds, and one was because of error in the peak detection step. The last false result and the column bleed artefact could be discarded when reviewing extracted ion chromatograms of the reported peaks (see Fig. 1). This emphasises that manual validation is important, especially at retention times with only one or a few signals. The impurities in the model compounds could be verified by comparison with data from pure standards of the model compounds. These results prove the method as they were truly unexpected to everyone involved in the project. In addition, peaks at unexpected masses at the same retention times as the spiked compounds were also observed. They were attributed as fragments and adducts of low intensity. That leaves only two false-positive results in the dead time region that could not be explained. For peaks appearing in the dead time region, different chromatography that gives retention to those compounds would yield a more informative positive result. This has, however, not been pursued. There were approximately 1,500 peaks per sample in the dataset. The risk of false reports increases with the size of the dataset. We are satisfied with the low number of falsely reported peaks in this concept study.

The method correctly reported all added model compounds that were found in the standards, including three impurities. To detect and gain information about a compound, it needs to be compatible with the chromatographic method and the ionisation method. To create a completely general detection method for unexpected contaminants, complementary analytical techniques need to be employed. The method can still be useful thanks to the wide range of compounds covered by electrospray ionisation in positive mode.

To find extreme concentrations of compounds that are present, a different detection criterion is needed: possible contaminants are identified as signals of higher intensity in the suspected sample than in the other samples. Such an analysis where each of the three brands, not including the spiked samples, is compared to the other two indicates that peak ratios greater than five or ten are rare (Table 4). Therefore, it seems likely that the approach can be used to detect if there is far too much of a food additive.

**Table 4:** The number of peaks from the one brand that has an average that is a factor (2, 5 or 10) times higher than the average from the other brands

Peak ratio	Number of peaks
2–5	26
5–10	1
>10	0

The results have been averaged over the three possible permutations

The approximately 1,500 peaks per sample were aligned to 3,686 unique peaks. Seventeen percent (609 peaks) existed in only one or two chromatograms. Given that the analyses were made in replicate, this means the dataset contained a lot of potential false-positive peaks. Luckily most of these were random noise, i.e. the peak did not exist in both the sample and the replicate. Furthermore, most of these peaks had a very low intensity. As a comparison, 70 % of these peaks had an intensity of less than one tenth of that of the main peak of aflatoxin B1, which was added in a concentration of approximately 400 ppb.

**Fig. 2:**
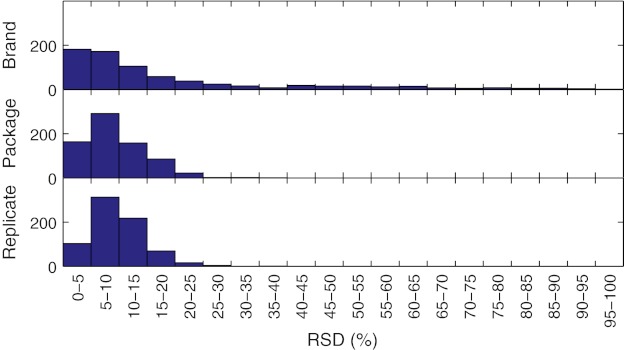
Histograms of the distribution of relative standard deviations of the aligned peaks

A nested analysis of variance (ANOVA) was used to assess the between-brand and experimental variability. Of the 892 peaks present in all the samples (excluding spiked samples), 90 showed significant between-brand differences. Figure 2 shows histograms of the relative standard deviation (RSD) distribution of the different levels of the nested ANOVA. The distributions within and between packages were almost identical. The RSD distribution between brands had a clear tail towards high values, composed mainly of the 90 peaks found to have statistical significance between brands. The RSD of the replicates (i.e. the within-package variability) mostly lay between 5 % and 15 % (see Fig. 2). High RSD values were predominantly associated with low intensity peaks.

Variability in the form of instrument drift in retention times are dealt with by the peak alignment, which relies on a peak detection step. A large amount of false peaks, i.e. noise detected as peaks by the peak detection algorithm, may decrease the quality of the alignment. Another issue is peaks not being detected. An alternative to the presented approach is to use warping on the full data [14]. Warping reduces the risk of misalignment as a result of errors in the peak detection, but has much higher computational complexity. Our results indicate that errors in the peak detection were not an important issue with respect to the aim of the study. Two modes of MS acquisition were compared, focus (resolution 40,000) and non-focus mode (resolution 20,000). In focus mode, smaller peaks can be detected, but the data is noisier resulting in higher proportion of noise being detected as peaks. A higher sampling frequency is likely to give improved peak detection, at the cost of noisier data. Depending on the application and on the sample, the sampling frequency could be set higher to reduce the amount of false peaks.

The extraction using 75 % acetonitrile and 1 % formic acid in water has been shown to be suitable for multi-residue LC–MS/MS (triple–quadrupole) analysis of drugs, pesticides, mycotoxins and other compounds [15] regardless of whether the compounds are acids, neutral molecules or bases [16]. The former study demonstrated that using this extraction mixture, recoveries were high and ion suppression effects low for most of the 109 compounds analysed simultaneously in a wide range of foods; altogether 19 matrices were analysed with analyte concentrations from 40 to 400 μg/kg. Thus, this mixture was expected to be suitable for extracting the initially unknown compounds in the present study. While these concentrations are higher than the limits of many compounds, the aim of the method is not to replace existing methods, but rather complement them to find unknown contaminants.

The method was originally developed for analysis in case of an incident. If a foodstuff starts to cause illness, the method can be used to find signals from additional chemical components compared to a prior or similar sample. These signals can then be used as a first step to identify the new compounds. For instance, if a brand of orange juice starts to cause illness, it can be compared to other brands of orange juice to determine whether any additional compounds are present which may have caused the illness. Once additional signals have been found, these compounds can be identified and their source can be located.

**Fig. 3:**
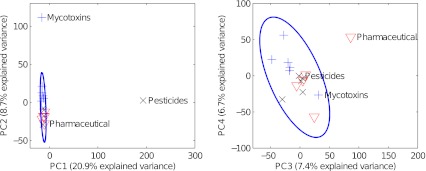
PCA score plot using aligned and scaled data. The first three components separate the three spiked samples from the rest of the dataset (+ Brämhult, × Tropicana, ∇ Willys)

We anticipate that a similar approach also can be used routinely for screening, batch control and similar analysis settings, for instance by using a multivariate control chart and determine presence of new peaks of significant intensity. Principal component analysis (PCA) of the aligned dataset revealed that the different brands did not form separate groups. While there are 90 peaks that vary significantly between the brands, their variance is masked by that of the other peaks in a mean-centred PCA. A PCA of the 90 significant peaks separates the three brands. However, we aim to find deviating analytes rather than to classify the brands. Low variance between the brands simplifies the detection of a potential new chemical hazard as a different brand could be used as a comparative sample with low risk of false positives. If the brands had formed separate groups, we might have found peaks unique to each brand. When the dataset was scaled by dividing each variable by its mean, the spiked samples separated into different principal components based on the number of added compounds (Fig. 3). Notably, data for the sample spiked with a single contaminant still clearly separated even though the chromatograms contained many false peaks compared to the number of peaks from the contaminant. This can be explained by the fact that peaks in the noise are not correlated, whereas sulfadoxin gave a total of 11 strongly correlated peaks. The correlation structure is captured by the PCA and that singles out the contaminated sample. The purpose of scaling the dataset was to determine whether the spiked samples could be separated. Intense peaks present in only one or a few samples dominate the variance and those samples are observed as outliers by extreme score values or high residuals. This kind of scaled PCA may be used for creating a sensitive multivariate control chart.

## Conclusions

The results demonstrate that it is possible to detect potential contaminants and, in general, non-targeted compounds in complex samples without prior knowledge of which compounds are present. All model compounds that were compatible with the LC–MS method were detected. We conclude that the method can be used to identify new or unexpected chemical hazards in food.

Technical replicates were critical for the data analysis. Because of the size of the dataset, several noise peaks were detected, but by comparing replicates, these could be discarded. Thus, the technical replicates act as a powerful filter to produce a more adequate peak list from one originating from an overly sensitive peak detection. The peak detection procedure requires an experienced analyst: parameter values need to be set so that all analyte peaks are detected but with as few false-positive results as possible.

The results of the data analysis suggested that there was little brand-to-brand variation between the three brands of orange juice tested. Future work will entail using the method to analyse datasets for more heterogeneous food matrices.
